# Fine-Tuning Modulation of Oxidation-Mediated Posttranslational Control of *Bradyrhizobium diazoefficiens* FixK_2_ Transcription Factor

**DOI:** 10.3390/ijms23095117

**Published:** 2022-05-04

**Authors:** Sergio Parejo, Juan J. Cabrera, Andrea Jiménez-Leiva, Laura Tomás-Gallardo, Eulogio J. Bedmar, Andrew J. Gates, Socorro Mesa

**Affiliations:** 1 Department of Soil Microbiology and Symbiotic Systems, Estación Experimental del Zaidín, CSIC, 18008 Granada, Spain; sergio.parejo@eez.csic.es (S.P.); juan.cabrera@eez.csic.es (J.J.C.); andrea.jimenez@eez.csic.es (A.J.-L.); eulogio.bedmar@eez.csic.es (E.J.B.); 2 Proteomics and Biochemistry Unit, Andalusian Centre for Developmental Biology, CSIC-Pablo de Olavide University, 41013 Seville, Spain; ltomgal@upo.es; 3 School of Biological Sciences, University of East Anglia, Norwich Research Park, Norwich NR4 7TJ, UK; a.gates@uea.ac.uk

**Keywords:** CRP/FNR proteins, in vitro transcription, microarrays, microoxia, protein–DNA interaction, rhizobia, symbiosis

## Abstract

FixK_2_ is a CRP/FNR-type transcription factor that plays a central role in a sophisticated regulatory network for the anoxic, microoxic and symbiotic lifestyles of the soybean endosymbiont *Bradyrhizobium diazoefficiens*. Aside from the balanced expression of the *fixK_2_* gene under microoxic conditions (induced by the two-component regulatory system FixLJ and negatively auto-repressed), FixK_2_ activity is posttranslationally controlled by proteolysis, and by the oxidation of a singular cysteine residue (C183) near its DNA-binding domain. To simulate the permanent oxidation of FixK_2_, we replaced C183 for aspartic acid. Purified C183D FixK_2_ protein showed both low DNA binding and in vitro transcriptional activation from the promoter of the *fixNOQP* operon, required for respiration under symbiosis. However, in a *B. diazoefficiens* strain coding for C183D FixK_2_, expression of a *fixNOQP’-‘lacZ* fusion was similar to that in the wild type, when both strains were grown microoxically. The C183D FixK_2_ encoding strain also showed a wild-type phenotype in symbiosis with soybeans, and increased *fixK_2_* gene expression levels and FixK_2_ protein abundance in cells. These two latter observations, together with the global transcriptional profile of the microoxically cultured C183D FixK_2_ encoding strain, suggest the existence of a finely tuned regulatory strategy to counterbalance the oxidation-mediated inactivation of FixK_2_ in vivo.

## 1. Introduction

Nitrogen (N) is an essential nutrient for all living organisms on Earth. Biological nitrogen fixation (BNF) and denitrification represent two crucial pathways in the biogeochemical N cycle, maintaining the global balance of combined N (reviewed in [1,2,3]). Rhizobia are important contributors to BNF, a process that is highly relevant for both agronomy and the environment, since it reduces the need for chemical fertilizers in agriculture. They consist of a large group of α- and β-proteobacteria that can establish symbiotic associations with leguminous plants (reviewed in [4]). Importantly, they express the nitrogenase enzyme, which catalyzes the reduction of N_2_ to ammonium inside nodules located at the roots and occasionally on the stems of the plant partner (reviewed in [5,6]). During the symbiotic interaction, rhizobia are challenged to respond and adapt their physiology to a battery of signals. These include oxidative stress generated by the plants or the low partial pressure of free oxygen within the nodules (microoxia) (reviewed in [6,7,8,9]). Microoxia is needed for the expression and functionality of nitrogenase and also the *cbb_3_*-type high-affinity terminal oxidase essential for bacterial respiration within the nodules (reviewed in [5,6,9,10,11]).

*Bradyrhizobium* species are the most widely employed diazotrophs as inoculants for soybean crops in agriculture [12]. In addition to being an efficient nitrogen fixer, *B. diazoefficiens* [13] is the only rhizobial species known for its ability to carry out complete denitrification, both in free-living and in symbiotic conditions ([14]; reviewed in [15,16]). In this bacterium, a complex regulatory network formed by two interconnected cascades (FixLJ-FixK_2_-NnrR and RegSR-NifA) controls the expression of genes required for microoxic, denitrifying and symbiotic modes of life ([17]; reviewed in [18]). The FixLJ-FixK_2_-NnrR cascade is oxygen-sensitive and activation by the two-component system FixLJ occurs at a concentration ≤ 5% O_2_, where the phosphorylated FixJ response regulator induces the expression of several genes, including *fixK_2_* (reviewed in [18]). The FixK_2_ protein plays a crucial role in this regulatory network, since it provides the link with the RegSR-NifA cascade and is also involved in the activation of hundreds of genes [19]. Among them, the *fixNOQP* operon encoding the high-affinity terminal oxidase *cbb*_3_ genes involved in structural and accessory components of denitrification or regulatory genes (e.g., *rpoN*_1_, *fixK_1_* and *nnrR*) are included.

FixK_2_ is a member of the cyclic AMP receptor protein (CRP)/fumarate-nitrate reductase regulator (FNR) superfamily of bacterial transcription factors, which includes proteins that respond unevenly to a wide spectrum of environmental and intracellular cues (reviewed in [20,21,22]). This class of proteins has been described to control functions such as photosynthesis, virulence, carbon source utilization, nitrogen fixation and various modes of respiratory electron transport. CRP/FNR-type regulators have a fairly low similarity, but retain a well-conserved domain structure. This common protein architecture comprises an amino-terminal sensing domain linked via a long α-helical region (required for dimerization of the active homodimer) to a helix-turn-helix (HTH)-type DNA-binding domain at the carboxy-terminus (reviewed in [21]). This HTH motif recognizes and interacts with a palindromic DNA sequence located at distinct coordinates within the promoter region of regulated target genes (reviewed in [20,21]). In the case of FixK_2_, the consensus DNA recognition sequence is an imperfect 14-base-pair palindrome (TTGA/C-N_6_-T/GCAA, FixK_2_ box) [23,24].

Within CRP/FNR-type proteins, the transcriptional output to environmental and intracellular stimuli results from the interaction between a signaling molecule and the sensing domain. This induces a conformational change required for productive binding of the active dimer to the recognition sequence located at regulated gene promoters (reviewed in [25]). Signal perception can be through a direct response via a chemical modification of the protein or by binding to a specific prosthetic group or an effector molecule (reviewed in [21]).

Unlike most of the CRP/FNR superfamily members, the existence of a cofactor in modulating FixK_2_ transcription activation is unknown. Instead, *fixK_2_*/FixK_2_ expression and FixK_2_ activity are subjected to complex transcriptional and posttranscriptional regulation (reviewed in [18]). Further to induction by the FixLJ system in response to microoxia, expression of the *fixK_2_* gene is auto-repressed by its own product by an as yet unidentified mechanism [26,27]. FixK_2_ is also controlled at a posttranslational level by oxidation [28] and by proteolysis, by both specific cleavage and also general degradation mediated by the ClpAP_1_ chaperone-protease system [29]. In addition, we recently observed that *fixK_2_* is among ~90 genes regulated at a posttranscriptional level in response to microoxia [30].

Oxidation-mediated posttranslational regulation of FixK_2_ occurs at the level of its single cysteine residue (C183), which resides in proximity to the DNA-binding domain [23,28]. The oxidation of this cysteine triggers protein inactivation either through the formation of dimers via an intermolecular disulfide bridge, or through the modification of cysteine to sulfenic, sulfinic or sulfonic acid variants, which inactivates the protein due to a steric hindrance effect and also to electrostatic repulsion with target promoters [23,28]. In vivo, FixK_2_ posttranslational oxidation might be relevant for the rapid cessation of transcriptional activity in response to reactive oxygen species (ROS) produced at several stages of the symbiotic interaction with soybeans (at the early stage of root hair infection, during endosymbiotic respiration and at late nodule senescence) (reviewed in [7,8,31]).

The aim of this work was to advance our understanding of the mechanism underpinning the oxidation-mediated posttranslational control of the FixK_2_ regulatory protein, both in vitro and in vivo. Our hypothesis was that if C183 in FixK_2_ was exchanged to aspartic acid, this semi-conservative replacement (due to both its size and charge) would permanently mimic FixK_2_ overoxidation (e.g., sulfenic/sulfinic acid cysteine derivatives). This stable modification might help to better unravel the consequences of FixK_2_ oxidation in vivo, especially regulation associated with transient bursts of ROS during symbiosis. We characterized the DNA-binding properties, in vitro transcription (IVT) activation activity and oligomeric state of recombinant C183D FixK_2_. The effect of C183D FixK_2_ was also analyzed in strains cultivated under free-living, microoxic conditions as well as in symbiosis with soybean plants. Together, our results reveal a fine-tuning mechanism in *B. diazoefficiens* to compensate for FixK_2_ inactivation in response to cellular oxidizing conditions.

## 2. Results

### 2.1. Assessing the Impact of C183D Exchange in FixK_2_ on In Vitro Transcription Activation Activity and Protein–DNA Interaction Ability

Transcriptional regulation mediated by the FixK_2_ protein is affected, among other factors, through an oxidation-mediated posttranslational control (reviewed in [18]). The C183 residue in FixK_2_ plays a central regulatory role because it is sensitive to ROS, giving rise to overoxidized species of the protein, i.e., sulfenic, sulfinic and sulfonic acid derivatives. In order to mimic FixK_2_ overoxidation, we performed a cysteine to aspartic acid replacement and subsequent functional analyses of the C183D FixK_2_ protein variant. In this context, its performance was compared with that of the genuine FixK_2_ protein [32], and with that of a C183S FixK_2_ derivative, which is oxidation-resistant [24]. All these proteins were previously purified as untagged variants using the intein-mediated purification with an affinity chitin-binding tag (IMPACT) methodology (New England Biolabs (NEB), Hitchin, UK).

**Figure 1:**
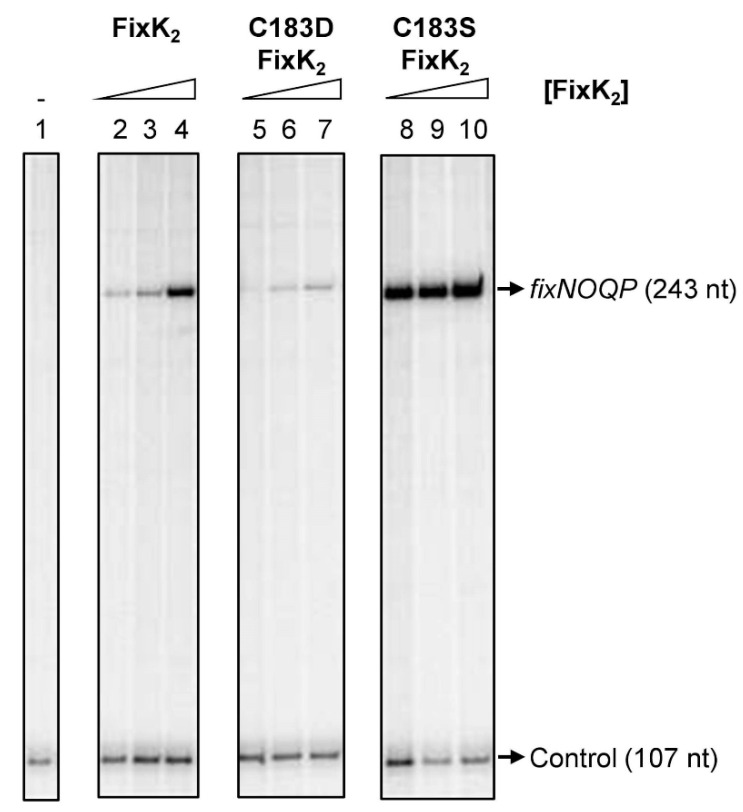
IVT activation from the *fixNOQP* promoter mediated by different FixK_2_ protein derivatives. Plasmid pRJ8816 harboring the *fixNOQP* promoter cloned upstream of the *B. diazoefficiens rrn* transcriptional terminator was employed as template for multiple-round IVT activation assays with *B. diazoefficiens* RNAP holoenzyme. A series of concentrations of FixK_2_ protein variants were added to the reactions: lane 1, no protein (-); lanes 2, 5 and 8, 0.5 µM; lanes 3, 6 and 9, 1.25 µM; lanes 4, 7 and 10, 2.5 µM. The positions of the *fixNOQP* transcript and the FixK_2_-independent transcript (used as control for the experiments) are depicted on the right. Each panel refers to different sections of the same gel. Shown are the results of a typical experiment that was performed at least twice. nt, nucleotides.

The ability of the C183D FixK_2_ protein to activate transcription in vitro in collaboration with *B. diazoefficiens* RNA polymerase (RNAP) was monitored in a multiple-round IVT activation assay using the template plasmid pRJ8816, which harbors the *fixNOQP* operon promoter cloned upstream of the *B. diazoefficiens rrn* transcriptional terminator (Figure 1) [33]. Importantly, this plasmid allows simultaneous analysis of both FixK_2_-dependent (*fixNOQP* transcript, 243 nucleotides [nt]) and FixK_2_-independent (control transcript, 107 nt) transcriptional responses elicited by *B. diazoefficiens* RNAP (Figure 1). The FixK_2_ protein efficiently activated transcription at 0.5 µM (Figure 1, lane 2), which increased at higher concentrations (Figure 1, lanes 3 and 4). In contrast, the C183D FixK_2_ derivative triggered low levels of transcription from the *fixNOQP* promoter even when 2.5 μM of the protein was present in the reaction (Figure 1, lane 7). However, the C183S FixK_2_ variant showed higher levels of transcription activation activity than the FixK_2_ protein, reaching saturation at 0.5 µM (Figure 1, lane 8).

**Figure 2:**
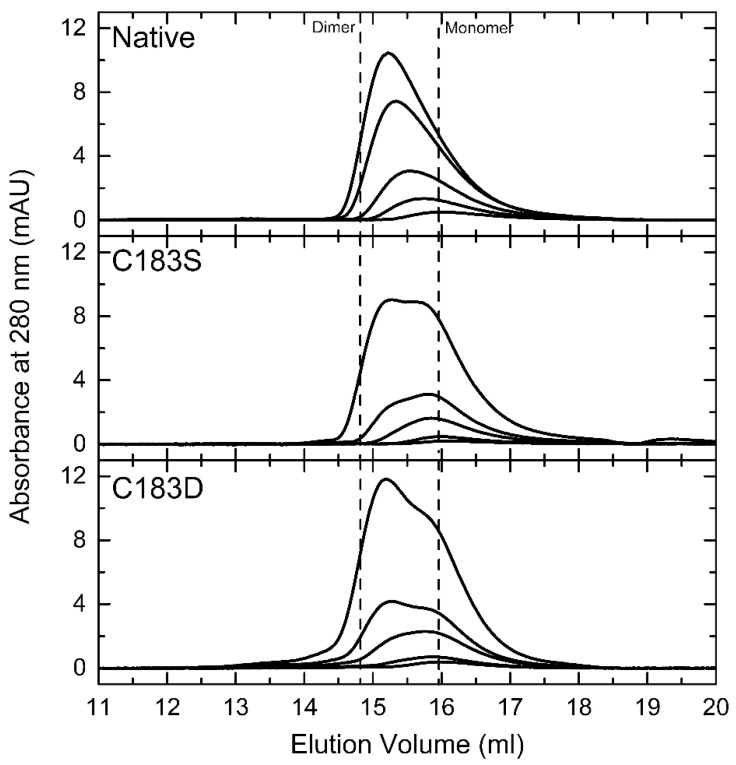
Comparative SEC of native FixK_2_ and C183S and C183D FixK_2_ variants at different protein concentrations. Elution profiles were monitored at 280 nm following chromatography of FixK_2_ loaded at 2.5, 5, 10, 20 and 30 µM (native, upper panel); C183S FixK_2_ at 2.5, 5, 10, 20 and 40 µM (C183S, middle panel); and C183D FixK_2_ at 2.5, 5, 10, 20 and 40 µM (C183D, bottom panel). The dashed lines show the calculated elution volume for the theoretical M_w_ of the monomeric (~26 kDa) and dimeric forms (~52 kDa).

Since FixK_2_ belongs to the CRP/FNR-type transcription factor family, which act as functional dimers, the solution oligomeric state of C183D FixK_2_ was analyzed by size-exclusion chromatography (SEC) and compared to those of native FixK_2_ and the C183S FixK_2_ derivative (Figure 2). These experiments were performed to determine whether the diminished transcription efficiency of the C183D FixK_2_ protein variant could be attributed to an altered oligomeric state. Importantly, prior to SEC, each protein derivative preparation was analyzed by denaturing sodium dodecyl sulfate polyacrylamide gel electrophoresis (SDS-PAGE) and showed a purity over ~95% for the band that corresponds to the predicted molecular mass of FixK_2_ (~25.6 kDa) (Figure S1). During non-denaturing individual SEC experiments for the three protein variants, chromatographic elution profiles showed a concentration-dependent behavior, with retention volumes ranging from the apparent molecular weight of the dimer (~52 kDa) to that of the monomer (~26 kDa) (Figure 2), as previously described for the N-terminally tagged wild-type protein [33]. The three proteins showed a monomer–dimer equilibrium; however, the proportion of the dimeric fraction with respect to the monomeric fraction was higher for the native FixK_2_ protein (Figure 2A) compared to C183S FixK_2_ and C183D FixK_2_ (Figure 2B and 2C, respectively) at similar concentrations. The reason for this difference might be related to the susceptibility of the wild-type derivative to the formation of disulfide bridges via C183. However, C183S FixK_2_ (Figure 2B) and C183D FixK_2_ (Figure 2C), which are devoid of cysteine residues, both showed similar monomer–dimer profiles despite their contrasting performance in transcriptional activation assays from the *fixNOQP* promoter. Therefore, the impaired IVT activation activity observed for the C183D FixK_2_ derivative is unlikely to be solely related to different oligomeric behavior.

**Figure 3:**
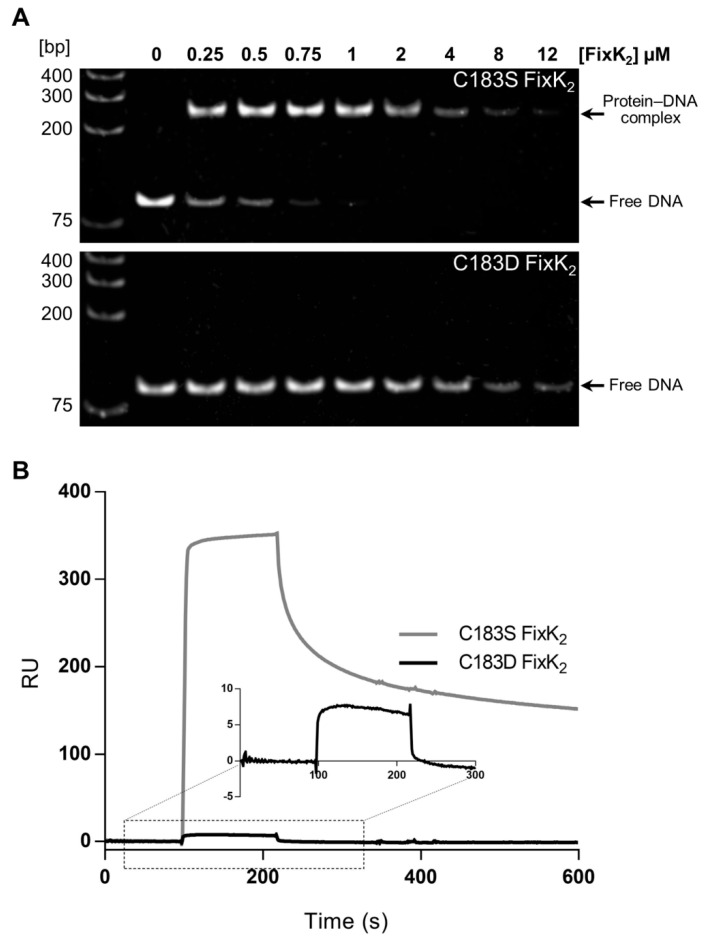
In vitro interaction of C183S and C183D FixK_2_ derivatives with the *fixNOQP* promoter tested by EMSA (**A**) and surface plasmon resonance (SPR) (**B**) approaches. (**A**) A 90-bp PCR fragment containing the FixK_2_ box at 20 nM was incubated with increasing concentrations (0 to 12 µM) of FixK_2_ protein variants, indicated at the top of each gel. Lower bands show free DNA, while upper bands correspond to the protein–DNA complexes. The molecular marker GeneRuler™ 1 Kb Plus DNA Ladder (Thermo Fisher Scientific, Waltham, MA, USA) is shown on the first lane. (**B**) A biotinylated double-stranded oligonucleotide containing the FixK_2_ box from the *fixNOQP* promoter was immobilized on a streptavidin (SA) sensor chip by biotin–streptavidin binding. The sensorgrams with the relative resonance units (RU) of the interaction with DNA of C183S and C183D FixK_2_ protein variants at 250 nM are shown. Data of the C183D FixK_2_ protein did not allow us to calculate any kinetic/affinity parameters.

To evaluate whether or not the C183D mutation in FixK_2_ affects the DNA-binding capacity of the protein, electrophoretic mobility shift DNA assays (EMSAs) were performed. Target DNA for these experiments was generated by PCR amplification of the promoter region of the *fixNOQP* operon. We found that a FixK_2_–DNA complex was readily detected when 0.25 μM of C183S FixK_2_ protein was included in the reaction (Figure 3A, gel at the top). However, a concentration at least 16-fold higher (i.e., 4 μM) of the C183D FixK_2_ protein was required to detect any interaction with DNA (Figure 3A, gel at the bottom), as determined by free-DNA disappearance, since the protein–DNA complexes apparently did not enter the gel at such protein concentration of this protein. Furthermore, a similar DNA mobility shift with each individual protein was only detected at a concentration approximately 32-fold higher of the C183D FixK_2_ protein (8 µM) with respect to the C183S FixK_2_ variant (0.25 µM) (Figure 3A), again determined by equivalent free DNA disappearance.

The DNA-binding properties of the C183D FixK_2_ variant were also determined by employing the surface plasmon resonance (SPR) methodology (Figure 3B). In these assays, the FixK_2_ box located within the *fixNOQP* promoter was immobilized on a streptavidin (SA) sensor chip and the binding kinetics and affinity were analyzed by monitoring the response in resonance units (RU) vs. time. In line with the EMSA results, purified C183D FixK_2_ interacted poorly with DNA (Figure 3B). Further, neither affinity nor kinetic parameters could be calculated as they were beyond the Biacore range and non-specific interactions were detected at high protein concentrations. This was in contrast with the results of a previous study performed with the C183S FixK_2_ derivative, which showed that FixK_2_–DNA interaction takes place at the nanomolar range and fitted well to a kinetic model for the interaction of one protein dimer per DNA molecule [24].

### 2.2. In Vivo Effects of C183 to Aspartic Acid Replacement in FixK_2_

**Figure 4:**
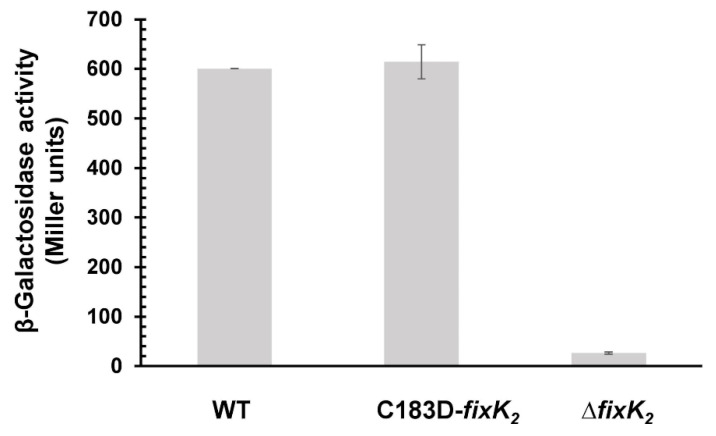
Expression data for a chromosomally integrated *fixNOQP’-’lacZ* fusion in different *B. diazoefficiens* backgrounds. Wild-type, C183D-*fixK_2_* and Δ*fixK_2_* strains were cultivated for 48 h microoxically (0.5% O_2_). β-Galactosidase values are means ± standard errors of a representative experiment performed with two parallel cultures assayed in quadruples. The experiment was repeated at least twice. WT, wild type.

To determine the effect of substituting C183 with aspartic acid in FixK_2_ in a cellular context, we performed a series of in vivo experiments. Firstly, we measured the β-Galactosidase activity of a chromosomally integrated *fixNOQP’-´lacZ* fusion in a *B. diazoefficiens* strain encoding C183D FixK_2_ (C183D-*fixK_2_*) compared to the wild-type and Δ*fixK_2_* strains, both used as controls (Figure 4). All strains were cultured under microoxic conditions (0.5% O_2_) for 48 h. An induction of approximately 600 Miller units (MU) was observed in the wild type, while, as expected, only basal levels were detected in the Δ*fixK_2_* strain (Figure 4). However, expression of the *fixNOQP´-´lacZ* in the C183D-*fixK_2_* strain was similar to that observed for wild-type cells, suggesting that, in vivo, other mechanisms counterbalance the impaired transcriptional output of the C183D FixK_2_ protein observed in vitro.

**Figure 5:**
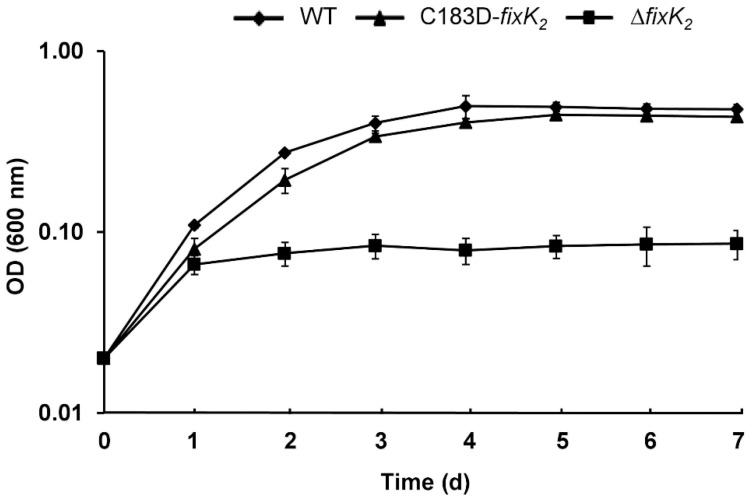
Denitrifying growth of the *B. diazoefficiens* C183D-*fixK_2_* strain (triangles). Wild type (WT, diamonds) and Δ*fixK_2_* (squares) were used as controls. Cells were grown anoxically with nitrate. Values ± standard errors are the mean of a representative experiment carried out with three parallel cultures. At least three replicates of the experiment were done.

Since FixK_2_ also directly or indirectly regulates the expression of genes involved in the denitrification process in *B. diazoefficiens* [19,34,35], we investigated whether the C183D mutation in FixK_2_ affects denitrifying growth (anoxia with nitrate as terminal respiratory electron acceptor) (Figure 5). Again, the C183D-*fixK_2_* strain showed growth profiles that were similar to the wild type rather than the Δ*fixK_2_* strain, where denitrifying growth is abolished.

The *fixNOQP* operon, employed as an archetypical target to monitor FixK_2_ activity [33], encodes the *cbb_3_* high-affinity terminal oxidase, required for bacterial respiration within root nodules. To investigate the ability of C183D FixK_2_ to support the plant-endosymbiotic interaction, we performed plant infection tests with soybeans inoculated with the wild type and the C183D-*fixK_2_* and Δ*fixK_2_* strains at two time-points: at 25 days post-inoculation (dpi), when maximal nitrogen fixation activity has been observed, and at 32 dpi, which corresponds to a late bacteroidal development stage [36] (Table 1).

No significant phenotypic differences, neither at 25 nor at 32 dpi, were observed in the C183D-*fixK_2_* strain compared to the wild type with regard to several parameters relevant for plant-endosymbiotic efficacy, such as shoot dry weight (SDW), nitrogen shoot content (N), nodule number per plant (NN), nodule dry weight per plant (NDW), dry weight per nodule (NDW/NN) and leghemoglobin content in nodules (Lb) (Table 1). This contrasted with the phenotype of the plants inoculated with the Δ*fixK_2_* strain, in which N, NDW/NN and Lb values were severely diminished (Table 1), which is in line with previous studies [24,26].

### 2.3. Appraisal of the Impact of the C183D Mutation on a Wider FixK_2_-Mediated Control Landscape

**Figure 6:**
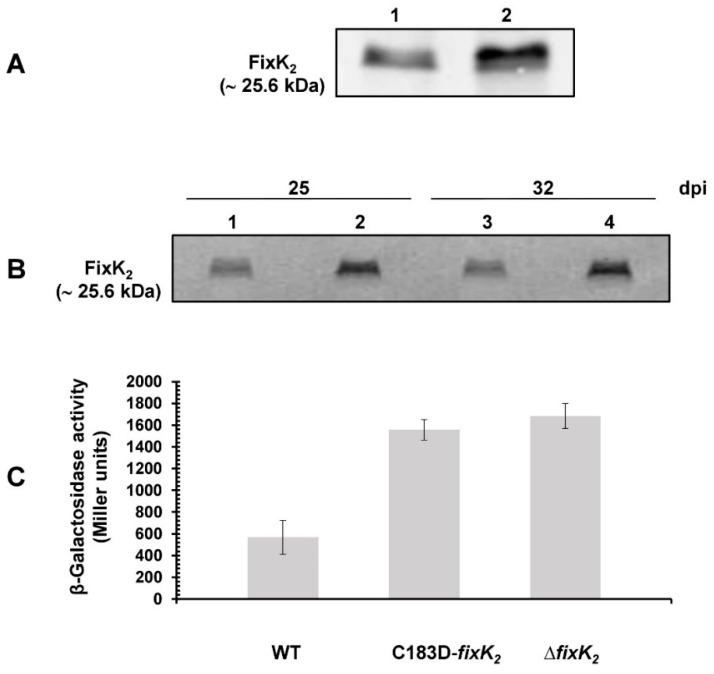
Expression of *fixK_2_* at protein (**A**,**B**) and transcriptional (**C**) levels. Steady-state levels of FixK_2_ protein in cells cultivated under microoxic free-living conditions (**A**) and in soybean bacteroids collected at 25 and 32 dpi (**B**). Immunodetection was performed with a polyclonal FixK_2_ antibody [28]. (**A**) 60 μg of crude extract of wild-type (lane 1) and C183D-*fixK_2_* strains (lane 2) both cultivated microoxically (0.5% O_2_). (**B**) 10 μL of soybean bacteroid crude extract of wild-type (lanes 1 and 3) and C183D-*fixK_2_* strains (lanes 2 and 4). Apparent molecular mass of FixK_2_ is shown on the left. Representative results of at least three independent biological replicates are shown. (**C**) β-Galactosidase activity from a chromosomally integrated *fixK_2_′-’lacZ* fusion in *B. diazoefficiens* wild-type, C183D-*fixK_2_* and Δ*fixK_2_* strains. Cells were cultivated for 48 h microoxically (0.5% O_2_). Values are the means ± standard errors of a typical experiment performed with two parallel cultures assayed in quadruples. The experiment was repeated at least twice. WT, wild type.

In order to reconcile and further understand the in vitro and in vivo results obtained with C183D FixK_2_, which suggested that, in cells, other mechanisms may compensate for the low DNA-binding capacity and IVT activation activity of the modified protein, a series of additional assays were performed. Firstly, we analyzed the abundance of FixK_2_ by Western blot of crude extracts from cells grown under microoxic free-living conditions and from soybean bacteroids (Figure 6A,B, respectively). Steady-state levels of FixK_2_ were approximately 2–3-fold higher in the C183D-*fixK_2_* strain than in the wild type (Figure 6A, lane 2 vs. lane 1). A similar profile was also observed in soybean bacteroids extracted from nodules at 25 and at 32 dpi (Figure 6B, lanes 2 and 4 vs. lanes 1 and 3, respectively).

Based on these results, we also monitored whether the C183D FixK_2_ modification affected the expression of the *fixK_2_* gene itself. Here, we measured β-Galactosidase activity from a *fixK_2_´-´lacZ* fusion integrated into the chromosome of the *B. diazoefficiens* C183D-*fixK_2_* strain when cultivated microoxically (Figure 6C). In line with the increased levels of FixK_2_ protein observed in the immunodetection experiments, expression of *fixK_2_* was around three-fold higher in the C183D-*fixK_2_* strain compared to those values observed in wild-type cells. This induction profile for the *fixK_2_′*-‘*lacZ* fusion was similar to that observed in the Δ*fixK_2_* strain (Figure 6C; [18,26,27]) and therefore indicated that de-repression of *fixK_2_* auto-regulation also occurred in the C183D-*fixK_2_* strain.

**Figure 7:**
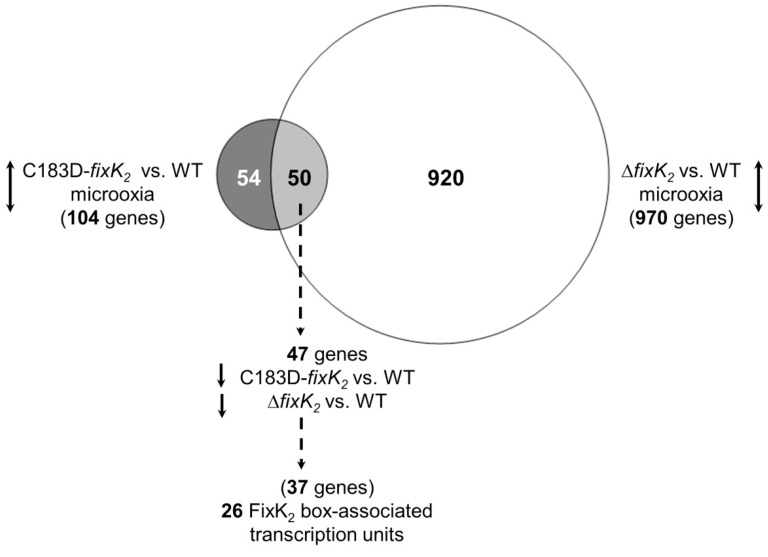
Workflow of microarray data analyses of the C183D-*fixK_2_* strain. Labels of the comparisons between specific transcription profiles are depicted alongside the circles. The total number of differentially expressed genes is indicated in parentheses. Up/down arrows refer to increased and decreased gene expression. The group of genes with differential expression in the C183D-*fixK_2_* strain (dark grey circle, left) showed an overlap of 50 genes (light grey circle, middle) with those in Δ*fixK_2_* strain (white circle, right; [19]), both grown microoxically (0.5% O_2_) and compared with the wild type grown in the same conditions. Within the overlap, 47 genes showed downregulated expression in both the C183D-*fixK_2_* and Δ*fixK_2_* strains, which includes 37 genes organized in mono- or polycistronic transcriptional units that harbor a putative FixK_2_ box within the promoter region (26 putative transcriptional units, see Table 2).

In order to examine whether other, more global mechanisms could be involved in the C183D FixK_2_ phenotype in vivo, a global transcriptional analysis of the *B. diazoefficiens* C183D-*fixK_2_* strain was performed and compared with that of the wild type, both grown under microoxic conditions. For this purpose, we employed the well-validated *B. diazoefficiens* custom-made GeneChip [37]. This comparative transcriptomic profile showed that 104 genes showed differential expression in the C183D-*fixK_2_* strain, with 26 genes being upregulated and 78 genes downregulated (Table S1, Datasheet A; Figure 7). As expected, we found the *fixK_2_* gene within the group of upregulated genes, and a relative change of fivefold was observed. However, among the downregulated genes in the C183D-*fixK_2_* strain background, a series of legitimate FixK_2_-activated targets such as *fixNOQP*, *fixGHIS* and *napEDABC* were not present. Similarly, the expression of genes encoding other CRP/FNR-type transcription factors under positive control of FixK_2_ (i.e., *nnrR*, *fixK_1_*, bll2109, bll3466) did not change.

The comparison of the C183D-*fixK_2_* strain profile with the previously published transcriptional data of the Δ*fixK_2_* strain under microoxic conditions [19] revealed a partial overlap between both groups of genes (Figure 7). In particular, while 54 genes, mainly represented by hypothetical and unknown proteins, were specific for the C183D-*fixK_2_* strain profile (Table S1, Datasheet B), a further group of 50 genes were present in both profiles (Figure 7; Table S1, Datasheet C). Of this subset, 47 genes were downregulated (i.e., activated by FixK_2_), and specifically, 37 of them were organized into 26 transcriptional units, with each harboring a putative FixK_2_ binding site (Figure 7; Table 2). Furthermore, this includes 10 genes belonging to the set defined as putative direct FixK_2_ targets [19], and, in particular, the *hspC2*, *ppsA*, *phaC2*, *hemN_2_* and bsr7087 genes, which were previously validated by IVT activation assays (compiled by Cabrera and coworkers [24]; Table 2). These observations demonstrate that the expression of certain FixK_2_-dependent targets is not counterbalanced in the C183D-*fixK_2_* strain background.

## 3. Discussion

FixK_2_ is one of 16 CRP/FNR-type proteins present in the genome of *B. diazoefficiens* [39] but is distinguished among this family of regulators since it is capable of activating the transcription of the genes it regulates in collaboration with the RNAP of *B. diazoefficiens* in vitro, without any identifiable effector molecule [33]. Alternatively, different levels of regulation have been described for FixK_2_: (i) it is integrated into a complex regulatory network that responds to low oxygen formed by two interlinked cascades (FixLJ-FixK_2_ and RegSR-NifA), where *fixK_2_* expression is balanced through FixLJ-mediated activation and FixK_2_-triggered auto-repression (direct or indirect by an unknown mechanism) ([17,19,26,27]; reviewed in [18]); (ii) the activity of FixK_2_ is modulated at posttranscriptional [30] and posttranslational levels (reviewed in [18]). This latter mode of regulation involves proteolysis by specific cleavage, and by general degradation mediated by the ClpAP_1_ chaperone-protease system [29] and oxidation at the level of residue C183 in response to oxidizing agents ([28]; reviewed in [18]).

Computational analyses of bacterial CRP/FNR family members performed by Matsui and coworkers [22] proposed that these proteins evolved from an ancestral FNR protein involved in nitrogen fixation. Although FixK-type proteins are part of the FNR group, they lack the [Fe-S] ligand-binding motif characteristic for FNR-type proteins. As with FixK_2_, other examples within the CRP/FNR protein family of regulatory proteins capable of activating gene transcription without the need of a cofactor are known and include (i) SdrP of *Thermus thermophilus* HB8, which is involved in the supply of nutrients and energy, redox control and the polyadenylation of mRNA. This protein not only is active in vitro without any cofactor but also lacks a putative binding pocket for a cofactor in its crystal structure [40]. (ii) PrfA of the human pathogen *Listeria monocytogenes* is capable of binding to its target DNA with low affinity without a cofactor [41] but its activity is modulated by carbon source availability in *L. monocytogenes* cells [42]. Recently, it was confirmed that reduced gluthatione is the ligand for PrfA, both in vivo and in vitro [43,44]. (iii) FNR of *Acidithiobacillus ferrooxidans* ATCC23270 has low affinity for its [Fe-S] cofactor to allow a better transition between both aerobic and anaerobic environments [45]. (iv) Vfr of *Pseudomonas aeruginosa* can activate the transcription of some of its target genes in the absence of a cofactor (reviewed in [46]). In addition to cofactor-mediated modulation, regulation of targets by *L. monocytogenes* PrfA depends on the steady-state levels of this transcription factor in cells, which is subject to transcriptional, translational and posttranslational control [47]. Similarly, the activity of *Escherichia coli* FNR has also been shown to be modulated by protein levels through the degradation of the monomeric apo-protein by the ClpXP proteolytic system under oxic conditions [48,49]. All these antecedents, together with the key role of FixK_2_ in the microoxic metabolism of *B. diazoefficiens*, both in free-living conditions and in symbiosis, as well as in denitrification [19,26,34], support the possibility of the existence of alternative mechanisms for this protein to respond to intracellular and environmental stimuli.

The crystal structure of C183S FixK_2_ in complex with its genuine DNA-binding site (FixK_2_ box) present at the promoter of the *fixNOQP* operon [23] revealed why the C183 residue of FixK_2_ plays such a key role in its posttranslational control by oxidation. This is due to its proximity to the DNA-binding domain and its susceptibility not only to the formation of disulfide bridges but also to the generation of overoxidized sulfenic, sulfinic and sulfonic acid species, which result in electrostatic repulsion and steric hindrance [28]. Specifically, C183 interacts directly with the adenine located in position 7 of strand W [23], which is located immediately before thymine in position 8, which establishes hydrophobic interactions with the L195 residue of the HTH DNA-binding motif of FixK_2_.

**Figure 8:**
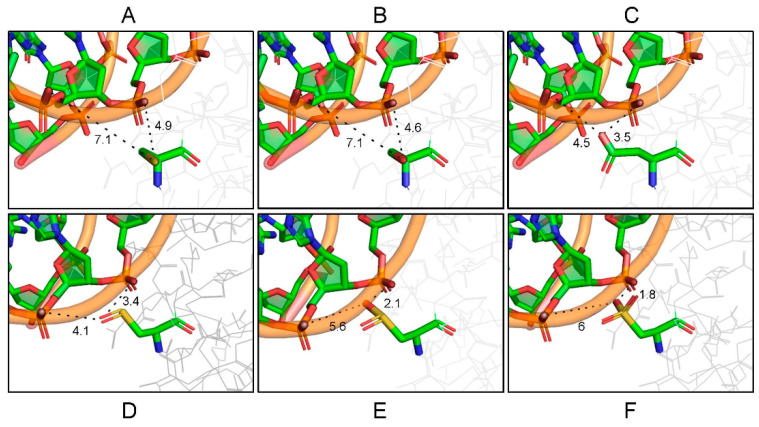
Modeling of different FixK_2_ protein variants with the double-stranded DNA containing the FixK_2_ box present at the *fixNOQP* promoter. Shown are the protein–DNA distances between the negatively charged oxygens of the phosphate group of the nitrogenous bases adenine 6 and adenine 7 of the strand W of DNA [23], and cysteine (**A**), serine (**B**), aspartic acid (**C**), cysteine–sulfenic acid (**D**), cysteine–sulfinic acid (**E**) and cysteine–sulfonic acid (**F**) residues of FixK_2_. The predictions of the 3D models of FixK_2_ and C183D FixK_2_ were obtained with the Pymol 2.2.3 program (https://pymol.org/2/; accessed on 13 October 2021), using the C183S FixK_2_–DNA structure as a template (http://wwpdb.org/; code 4I2O; accessed on 7 June 2021). Visualization of molecular structures and interactions was performed using the Discovery Studio Visualizer program, version V20.1.0.19295 (BIOVIA, Waltham, MA, USA), which also allowed the modeling of sulfenic, sulfinic and sulfonic acid derivatives of FixK_2_. Distances in angstroms (Å) are represented by dashed lines; adenine 6 on the left; adenine 7 on the right.

In our work, we have analyzed whether the exchange of C183 for an aspartic acid residue can simulate the permanent oxidation of FixK_2_. To explore how the C183D mutation may affect FixK_2_–DNA interaction in silico, we modeled a battery of protein derivatives (i.e., FixK_2_, C183S FixK_2_, C183D FixK_2_ and the sulfenic, sulfinic and sulfonic FixK_2_ variants) with the double-stranded FixK_2_ box DNA sequence from the *fixNOQP* promoter (Figure 8). According to these predictions, the replacement of C183 by aspartic acid causes the acquisition of a free negative charge and consequently an electrostatic repulsion with the phosphate groups of both the adenine 6 and adenine 7 bases of strand W of the target DNA [23]. Furthermore, the presence of the oxygen atom from the aspartate branched side chain also gives rise to steric hindrance due to the proximity of this atom to the bases described above, reducing the intermolecular distances of 7.1 and 4.9 angstroms (Å), to 4.5 and 3.5 Å, respectively (Figure 8A and Figure 8C). Thus, the C183D FixK_2_ derivative–DNA interaction likely mimics that of the sulfenic-derived cysteine (Figure 8D) and the sulfinic-derived cysteine (Figure 8E) due to the size and charge of each radical, respectively, rather than the most oxidized sulfinic-derived cysteine (Figure 8F).

The oxidation-mediated FixK_2_ inactivation similarity of the C183D FixK_2_ derivative was first analyzed in vitro. As expected, purified C183D FixK_2_ showed a low DNA-binding ability, determined by both SPR and EMSA approaches (Figure 3). This may also have affected the interaction with the RNAP polymerase and holocomplex conformation required for transcriptional output, as an impaired IVT activation capacity (a reduction of approximately 75%) for the C183D FixK_2_ protein derivate was observed in comparison to the FixK_2_ and C183S FixK_2_ variants (Figure 1). Furthermore, the monomer–dimer equilibrium of the oligomeric state of the C183D FixK_2_ protein variant appeared to be shifted more to the monomeric form in comparison to that of the native FixK_2_ protein (Figure 2). However, as this profile was fairly similar to that of the oxidation-insensitive C183S FixK_2_ protein, which interacts effectively with DNA and is fully active (Figure 1), it cannot be taken as the main factor to explain its deficiency in both DNA-binding capacity and IVT activation activity.

Despite the results found in vitro, intriguingly, the *B. diazoefficiens* C183D-*fixK_2_* strain showed a wild-type phenotype with regard to the expression of a *fixNOQP’-‘lacZ* fusion under microoxic conditions (Figure 4), its denitrifying growth behavior (Figure 5) and its symbiotic performance with soybeans (Table 1). This was in contrast with the phenotype of a Δ*fixK_2_* strain [24,26] and indicated the existence of alternative mechanisms in *B. diazoefficiens* cells, which compensates for the in vitro characteristics of the C183D FixK_2_ protein variant. To test this hypothesis, we then monitored the steady-state levels of C183D FixK_2_ protein in both *B. diazoefficiens* cells grown under free-living microoxic conditions and in soybean bacteroids isolated from nodules at 25 and 32 dpi. In all conditions tested, the abundance of the C183D FixK_2_ protein was higher (approximately 2–3 fold) than the wild-type protein (Figure 6), which could explain the absence of a phenotype of the *B. diazoefficiens* C183D-*fixK_2_* strain in our in vivo assays.

In order to obtain a global overview of the effect of the C183D replacement in FixK_2_, a transcriptomic profile of the *B. diazoefficiens* C183D-*fixK_2_* strain grown under microoxic conditions was next performed and compared to that of the wild type. Some remarks are here mentioned. A high proportion (920 out of 970) of the genes belonging to the FixK_2_ regulon did not show differential expression in the C183D-*fixK_2_* strain (Figure 7). This group includes other genes encoding CRP/FNR-type regulators whose expression is activated by FixK_2_, such as bll2109, bll3466, *fixK_1_* and *nnrR* [19]. This finding, together with the increased abundance of the C183D FixK_2_ protein (Figure 6), might be the rationale for the compensated expression of genes belonging to the FixK_2_ regulon in the C183D-*fixK_2_* strain. In this context, we should not overlook that the C183D FixK_2_ variant did not seem to mimic the inactive, most oxidized, sulfonic acid derivative of the FixK_2_ protein (Figure 8), which might contribute to the mild phenotype of the *B. diazoefficiens* C183D-*fixK_2_* strain.

Regardless of these arguments, 104 genes still showed differential expression in the C183D-*fixK_2_* strain in comparison with the wild type. Interestingly, 47 genes belonging to this group are under the positive control of FixK_2_, and 37 of them are organized in 26 transcriptional units that contain a FixK_2_ binding site within their promoter region (Figure 7; Table 2). This set includes direct targets compiled in [24], such as *hemN_2_*, *phbC2*, *ppsA*, blr4637 or bsr7087, but neither the *fixNOQP* operon encoding the *cbb_3_* high-affinity terminal oxidase nor the *napEDABC* genes encoding the periplasmic nitrate reductase involved in denitrification were present. These observations indicate that the overexpression of C183D FixK_2_ is not sufficient to compensate for the FixK_2_-mediated activation of transcription for all its targets. However, the inspection of the FixK_2_ boxes associated with the 26 transcription units, as well as the neighbor nucleotides (positions 6 and 7 of strand W of the *fixNOQP* promoter DNA; [23]), did not reveal a conserved pattern that could offer a plausible reason for this differential behavior of the C183D FixK_2_ protein with respect to the activation of the expression of direct targets.

Within the group of genes differentially expressed in the C183D-*fixK_2_* strain, around half (54 out of 104; Table S1) were not part of the FixK_2_ regulon. Among them, we did not find induction of those encoding other CRP/FNR-like proteins that could also counterbalance the constrained behavior of the C183D FixK_2_ variant. Instead, we encountered a large proportion of genes that code for hypothetical or unknown proteins, which makes it difficult to conduct a more comprehensive study.

Importantly, in accordance with the *fixK_2_’-’lacZ* fusion data determined under microoxic conditions (Figure 6), we found increased expression of the *fixK_2_* gene in the C183D-*fixK_2_* strain in comparison with the wild type. This enhanced expression was also previously found in the Δ*fixK_2_* strain [18,26,27], which is an indication that FixK_2_ negatively regulates its own expression (directly or indirectly) by an unknown mechanism. Reutimann and coworkers [27] proposed that this control is likely indirect, where the FixK_2_ protein may be involved in the activation of its own repressor or an activator of the *fixK_2_* repressor gene. As de-repression of the *fixK_2_* gene still occurred in the C183D-*fixK_2_* strain, we surveyed the list of genes that appeared to be downregulated in both C183D-*fixK_2_* and Δ*fixK_2_* regulons to identify possible candidates. None of the remaining regulatory genes previously proposed (i.e., blr1216, bsr4636, blr7666) ([27]; reviewed in [18]) appeared in such groups of genes (Table S1). Nevertheless, we found a predicted response regulator gene, bll0330, which harbors a putative FixK_2_ binding site within its promoter region (Table 2). Although its expression is also under the positive control of the response regulator FixJ, it was previously overlooked as it is not induced under microoxic conditions [19]. The functional analysis of this gene in the context of *fixK_2_* negative auto-regulation would be interesting to pursue; however, we believe that this is beyond the scope of this paper.

## 4. Materials and Methods

### 4.1. Strains, Plasmids, and Primers

A detailed description of the plasmids and bacterial strains used in this work is compiled in Table 3. Table S2 describes primer names and sequences employed in this study.

### 4.2. Media and Growth Conditions

*E. coli* cells were typically grown in Luria–Bertani (LB) medium [55] at 37 °C overnight. When needed, antibiotics were added at the following concentrations (in µg·mL^−1^): ampicillin, 200; kanamycin, 30; spectinomycin, 25; streptomycin, 25; tetracycline, 10.

*B. diazoefficiens* strains were routinely cultured oxically at 30 °C under rigorous shaking (170 rpm) in a peptone–salt–yeast extract (PSY) medium [19,51]. Microoxic cultures (0.5% O_2_ in PSY medium), and under denitrifying conditions (anoxia in yeast extract–mannitol [YEM] medium supplemented with 10 mM KNO_3_; [56]) were essentially prepared as described previously [24]. The initial optical density (OD) at 600 nm of the cultures was 0.02, except for those employed in β-Galactosidase assays, which was 0.2, since not all the strains showed the same growth behavior. In the microoxic cultures, the gas phase was exchanged in cycles of 8/16 h. Antibiotic concentrations in *B. diazoefficiens* cultures were as follows (in μg·mL^−1^): chloramphenicol, 15 (solid medium); kanamycin, 200 (solid medium), 100 (liquid medium); spectinomycin, 200 (solid medium), 100 (liquid medium); tetracycline 100 (solid medium), 50 (liquid medium).

### 4.3. Strain and Plasmid Construction

A *B. diazoefficiens* strain that encodes a C183D FixK_2_ protein variant was constructed using a markerless mutagenesis approach based on the *sacB*-based methodology [54,57]. Firstly, C183 in FixK_2_ was exchanged by aspartic acid using site-directed mutagenesis and plasmid pRJ8848 as a template, and oligonucleotides fixK_2__mut59 and fixK_2__mut60, yielding plasmid pMB1250. A 1.843-kb *Bam*HI/*Xba*I fragment from pMB1250 was then cloned into the corresponding sites of the pBBR1MCS-2 vector, thus resulting in plasmid pMB1251. Next, a 3.965-kb *Not*I fragment from pMB1251 was inserted into the linearized *Not*I pRJ9041 plasmid, to give rise to plasmid pMB1256. This plasmid was subsequently cut with *Bgl*II, and recirculation of a 4.974-kb fragment yielded plasmid pMB1254. Finally, to construct plasmid pMB1255, a 1.849-kb *Bam*HI fragment derived from plasmid pMB1254 was cloned into the suicide vector pK18*mobsacB*. Plasmid pMB1255 was then transferred to *E. coli* S17.1 cells, which were employed in biparental conjugation with *B. diazoefficiens* wild type. Single recombination transconjugants were selected by kanamycin resistance, followed by double recombination selection by sucrose resistance, as described elsewhere [57]. The genomic organization of the resulting markerless strain encoding a C183D FixK_2_ derivative (strain 1255) was verified by PCR and sequencing using specific primers (Table S2).

In order to construct a plasmid that expresses a C183D FixK_2_ derivative fused at its C-terminal region with the *Mxe* GyrA-intein–chitin-binding domain (CBD) expressed under the control of the T7 promoter, a 727-bp PCR-amplified fragment from pMB1251 with the oligonucleotides fixK_2__mut19 and fixK_2__mut58 was restricted with *Nde*I and *Spe*I and subsequently cloned in frame into the pTXB1 vector (NEB, Hitchin, UK), thus resulting in plasmid pMB1253. The correctness of the plasmid pMB1253 sequence was verified by sequencing with suitable primers (Table S2).

To construct *B. diazoefficiens* C183D FixK_2_ encoding strains harboring either a *fixNOQP’-‘lacZ* or a *fixK_2_′-‘lacZ* translational fusion, plasmids pRJ3603 and pMB1109 were transferred from *E. coli* S17.1 cells via biparental conjugation into the chromosome of the 1255 strain. Transconjugants were selected by tetracycline resistance and further verified by PCR and sequencing, yielding strains 1255-3603 and 1255-1109, expressing *fixNOQP’-‘lacZ* and *fixK_2_′-‘lacZ* fusions, respectively.

Plasmid and genomic DNA isolation was performed using the Qiagen Plasmid Kit (Qiagen, Germantown, MD, USA) and REALPURE Genomic DNA (Durviz, Valencia, Spain), respectively.

### 4.4. β-Galactosidase Activity Assays

Expression of *fixNOQP’-‘lacZ* and *fixK_2_′-‘lacZ* fusions in *B. diazoefficiens* cells grown under microoxic conditions was analyzed by measuring β-Galactosidase activity. Cells cultivated for 48 h were first permeabilized and subsequently used for the assays, as previously described [55,57]. The absorbance at 420 nm of the enzymatic reactions and at 600 nm of the cultures was recorded in a plate reader (SUNRISE Absorbance Reader; TECAN, Männedorf, Switzerland) using the XFluor4 software (TECAN, Männedorf, Switzerland). These data were used to calculate the specific activity of β-Galactosidase in Miller units (MU).

### 4.5. Plant Infection Test and Physiological Analyses

Plant inoculation and growth experiments were performed essentially as described previously [58]. Soybean seeds (*Glycine max* L. Merr., cv. Williams 82, harvest at October 2011) were firstly surface-sterilized and germinated at 30 °C for 48 h in darkness. After germination, seeds were sown in 0.25 L pots containing sterile vermiculite and 50 mL of modified Jensen N-free solution, as indicated earlier [58]. The seedlings were then inoculated independently with cell suspensions of each strain in sterile saline solution (0.9% *w*/*v* NaCl) at an OD_600_ of 0.5 (~10^5^ cells mL^−1^), prepared from oxically grown cultures collected at stationary phase (OD_600_~1). Plants were then cultivated under controlled conditions with an initial irrigation with modified Jensen medium followed by sterile deionized water until harvest at 25 and 32 dpi.

The plant physiology parameters nodule number per plant (NN), nodule dry weight (NDW) per plant, dry weight per nodule (NDW/NN) and shoot dry weight (SDW) were measured after harvesting as described by Tortosa and coworkers [58]. For bacteroid isolation and additional analyses, a minimum of 1 g of fresh nodules randomly collected from at least 3 plants were stored at −80 °C after quick freezing in liquid nitrogen. SDW was recorded after 3 days at 70 °C; samples were ground to less than 0.5 mm for nitrogen (N) determination. N content in SDW was measured by the Dumas method using the LECO TruSpec CN Elemental Analyzer [59].

For leghemoglobin (Lb) determination in the nodular fraction, 0.5 to 1.0 g nodules were manually homogenized by using a cooled porcelain pestle and mortar with 6 mL of buffer solution (50 mM Na_2_HPO_4_ · 2H_2_O/NaH_2_PO_4_ · 2H_2_O, pH 7.4, 0.02% *w*/*v* K_3_Fe(CN)_6_, and 0.1% *w*/*v* NaHCO_3_) and 0.1 g of polyvinyl poly(vinlylpolypyrrolidone) (PVPP) according to the methodology described in previous studies [58]. Then, the extract was centrifuged at 12,000× *g* at 4 °C for 20 min. Lb content was fluorometrically determined after an acidic reaction at 120 °C during 30 min, according to LaRue and Child [60]. After cooling the samples, the fluorescence in each tube was measured with a spectrophotofluorometer (Shimadzu Scientific Instruments, Kyoto, Japan) (λ_excitation_ = 405 and λ_absorption_ = 600 nm). Non-autoclaved tubes containing acidic nodular fraction were used as a control.

### 4.6. Overexpression and Purification of Non-Tagged FixK_2_ Protein Variants

Non-tagged FixK_2_ protein derivatives were purified with the IMPACT system (NEB, Hitchin, UK) according to the protocol detailed in [24]. In brief, *E. coli* ER2566 cells individually transformed with plasmids pRJ0051, pRJ0053 and pMB1253 were grown in 500 mL of LB medium at 37 °C until an OD_600_ of 0.3. The cultures were then incubated for 1 h at 30 °C up to an OD_600_ of 0.8, before addition of 0.1 mM IPTG for the induction of overexpression of the individual recombinant proteins. After incubation for 16 h at 16 °C, cells were collected and employed for protein purification [24]. Fractions of the different purification steps were collected and analyzed by Blue Coomassie-stained 14% SDS-PAGE, as described by Laemmli [61]. Cell pellets were resuspended in loading dye (62.5 mM Tris HCl pH 6.8, 2% SDS, 10% glycerol, 50 mM dithiothreitol [DTT], 0.01% bromophenol blue) in a proportion of 100 μL per mL of OD_600_ = 1 and subsequently boiled at 95 °C for 10 min and centrifuged at 12,000× *g* for 5 min before loading. For desalting, protein fractions from the affinity chromatography were pooled and buffer-exchanged by passing them through a prepacked Sephadex G-25M column (PD-10; Cytiva Europe GmbH, Cornellá de Llobregat, Spain) equilibrated with the suitable buffer for each further assay (IVT activation activity, SEC, EMSA, SPR).

### 4.7. In Vitro Transcription Activation Assay

IVT activation experiments were basically performed as described in previous studies [24,33,62]. Essentially, 20 μL reactions containing the basic transcription components, 1 μg of *B. diazoefficiens* RNAP, 750 ng of plasmid pRJ8816 that harbors the promoter of the *fixNOQP* operon [33] and different concentrations (0, 0.5, 1.25 and 2.5 μM) of individual protein derivatives (i.e., FixK_2_, C183S FixK_2_ and C183D FixK_2_) were incubated at 37 °C for 30 min. Transcription products were monitored with a PhosphorImager (Molecular Dynamics, Massachusetts, MA, USA) and signal intensities were evaluated with the Image Lab^TM^ software (Bio-Rad, California, CA, USA).

### 4.8. Size-Exclusion Chromatography Experiments

Analytical SEC experiments of the FixK_2_ protein derivatives were performed at room temperature on a Superdex 200 10/300 GL column (Cytiva, Little Chalfont, UK) using an ÄKTA PURE protein purification system (Cytiva, Little Chalfont, UK). After equilibrating the column with elution buffer (40 mM Tris-HCl, pH 7.0, 150 mM KCl 0.1 mM EDTA), 100 µL protein samples were injected and separated at a flow rate of 0.75 mL.min^−1^. Absorbance was recorded at 280 nm. The following proteins were used as standards for calibration (Figure S2): conalbumin (75 kDa), ovalbumin (43 kDa), carbonic anhydrase (29 kDa), ribonuclease A (13.7 kDa) and aprotinin (6.5 kDa) (Cytiva, Little Chalfont, UK). Gel filtration experiments were repeated at least three times with independent preparations of each protein at a range of at least five concentrations. The UNICORN™ system control software (Cytiva, Little Chalfont, UK) was employed to program the chromatography runs and for preliminary analyses of the data by adjusting for injection times.

### 4.9. Electrophoretic Mobility Shift DNA Assays

Stable FixK_2_–DNA interaction was tested electrophoretically. First, 15 µL reactions containing 10 ng of purified 90-bp PCR fragment spanning the promoter region of the *fixNOQP* operon (Table S2) and different protein concentrations, from 0 to 12 µM, in modified IVT buffer (40 mM Tris-HCl pH 8, 10 mM MgCl_2_, 0.1 mM EDTA, 0.1 mM DTT, 150 mM KCl, 0.4 mM K_3_PO_4_), were incubated for 30 min at room temperature. Reactions were mixed with one sixth volume of loading dye (30% glycerol in modified IVT buffer supplemented with bromophenol blue) and loaded onto a 6% non-denaturing polyacrylamide–0.5X Tris-Borate EDTA (TBE) gel. After running the electrophoresis for 40 min at 180 V, gels were incubated in a 1X SYBR-Gold (Invitrogen, Waltham, MA, USA) solution in 0.5X TBE for 30 min. Finally, UV-induced signals were detected by a Gel Doc XR+ System (Bio-Rad, California, CA, USA) and quantified with the Quantity One and Image Lab software (Bio-Rad, California, CA, USA).

### 4.10. Surface Plasmon Resonance Analyses

FixK_2_–DNA interaction ability was analyzed by SPR using a Biacore X100 Biosensor (Cytiva Europe GmbH, Cornellá de Llobregat, Spain) with SA sensor chips according to the methodology described by Cabrera and coworkers [24]. All buffers were previously filtered and degassed. The biotinylated double-stranded *fixNOQP* promoter region was synthesized by annealing complementary primers (Table S2), leaving the biotinylated primer at 10 µM. Then, the double-stranded oligonucleotide was diluted at 5 nM in immobilization buffer (Tris-HCl 10 mM pH 7.5, 50 mM NaCl, 1 mM EDTA) and captured at 100 RU in a sensor chip. Protein–DNA interaction assays were carried out in running buffer (40 mM Tris-HCl pH 7.0, 150 mM KCl, 0.1 mM EDTA) supplemented with 0.005% Tween 20 at 25 °C. The analyte was injected in both flow cells at 40 μL/min during 120 s of contact time, followed by 120 s of dissociation. In a first round, the analyte was diluted in running buffer from 0 to 250 nM in a random order, with at least one duplicate of a low concentration analyte after a higher concentration. Range of protein concentration was extended up to 3 μM in further experiments. The sensor surface was regenerated with injections of 0.2% SDS at 30 μL/min during 60 s. The number of trials, computer support and data analysis and quantification were as described earlier [24].

### 4.11. Immunoblot Detection of FixK_2_

Steady-state levels of FixK_2_ protein were monitored in *B. diazoefficiens* cells grown under microoxic conditions and in soybean bacteroids by immunoblotting using a polyclonal antibody against FixK_2_ [28]. At least three biological replicates of 300 mL of microoxically grown cultures (0.5% O_2_) at mid-exponential phase (OD_600_ of 0.45–0.58) were collected (5000× *g*, 7 min, 4 °C), washed with fractionation buffer (40 mM Tris-HCl pH 7.0, 150 mM KCl) and resuspended in 1.5 mL of the same buffer containing 0.2 mM 4-[2-Aminoethyl] benzenesulfonyl fluoride hydrochloride (AEBSF). Cell suspensions were disrupted by three passes through a cold French pressure cell (SLM Aminco, Jessup, MD, USA) at approximately 120 MPa, and subsequently centrifuged (27,000× *g*, 30 min, 4 °C) to obtain total cell-free extracts.

Isolation of bacteroids from soybean nodules inoculated with the different strains was performed as described elsewhere [14,58]. In short, 0.8 to 1 g of nodules per strain and condition were employed. After extraction, bacteroids were resuspended in 2 mL of 50 mM Tris-HCl pH 7.4. Cell density of bacteroid suspensions was determined and adjusted to an equal OD_600_ with the same buffer. Then, aliquots were taken, centrifuged at 12,000× *g* for 5 min and resuspended in six-fold-diluted SDS loading dye (350 mM Tris-HCl pH 6.8, 10% SDS, 30% glycerol, 620 mM DTT, 0.01% bromophenol blue) in a proportion of 20 μL per mL of OD_600_ = 1. Finally, they were boiled at 95 °C for 10 min and centrifuged at 12,000× *g* for 5 min before loading.

Conditions of SDS-PAGE and Western blotting were similar to those described in previous studies [30,62]. Samples were resolved in 14% SDS-PAGE, and subsequently transferred to nitrocellulose membranes using a Trans-Blot Turbo System (Bio-Rad, California, CA, USA). A rabbit-derived polyclonal antibody against FixK_2_ [28] at a 1:1000 dilution was used as primary antibody, while a horseradish peroxidase (HRP)-conjugated goat anti-rabbit IgG (Bio-Rad, California, CA, USA) at a 1:3500 dilution was employed as secondary antibody. Visualization of the signals was performed with a ChemiDoc XRS instrument (Universal Hood II, Bio-Rad California, CA, USA). The Quantity One and Image Lab software programs (Bio-Rad, California, CA, USA) were employed for image analyses.

### 4.12. Determination of Protein Concentration

Protein concentrations of samples employed in Western blot assays, as well as of purified recombinant proteins, were determined using the Bio-Rad reagent (Bio-Rad, California, CA, USA) and bovine serum albumin (BSA) as the standard protein for the calibration curve. The concentration of purified proteins used in this study is referred to in the dimeric form.

### 4.13. Microarray Sample Preparation and Data Analyses

For microarray experiments, *B. diazoefficiens* cultures were grown to mid-exponential phase (OD_600_ of 0.45 to 0.58). Cell harvest, isolation of total RNA, cDNA synthesis, fragmentation, labeling and conditions for hybridization with a custom-designed *B. diazoefficiens* Gene Chip BJAPETHa520090 (Affymetrix, Santa Clara, CA, USA) were as described in previous studies [19,36,37].

For these experiments, 1.8 µg of labeled fragmented cDNA was hybridized to the arrays. A minimum of three independent biological samples were analyzed. Signal intensities detection, normalization and analyses were done with Affymetrix Expression Console software version 1.4.1 (Affymetrix, Santa Clara, CA, USA). Transcriptome analysis Console 3.1 software (Affymetrix, Santa Clara, CA, USA) was used for comparative analyses. Normalized intensities (MAS 5.0 algorithm) were compared between conditions using one-way between-subject ANOVA (ANOVA *p*-value < 0.05). Only genes that passed the statistical tests and where the change in expression (measured as *n*-fold change [FC]) was ≥2 or ≤−2 in comparisons between two strains were considered as differentially expressed.

### 4.14. Biocomputing Analyses

In silico analyses of the interaction of the battery of FixK_2_ protein derivatives with DNA were performed based on the structure of the FixK_2_–DNA complex ([23]; http://wwpdb.org/, entry PDB 4I2O; accessed on 7 June 2021). The prediction of the 3D models of FixK_2_ and C183D-FixK_2_ was obtained with the Pymol 2.2.3 program (https://pymol.org/2/; accessed on 13 October 2021), using the C183S FixK_2_–DNA structure as a template. The visualization of molecular structures and interactions was performed using the Discovery Studio Visualizer program version V20.1.0.19295 (BIOVIA, Waltham, MA, USA), which also allowed modeling and predictions with derivatives of FixK_2_ proteins that harbor specific mutations or alterations in the oxidation state.

## 5. Conclusions

The main goal of this work was to better understand FixK_2_-dependent regulation essential for low-oxygen metabolism (microoxia) of the model denitrifying plant-endosymbiotic bacterium *B. diazoefficiens*. Microoxia has been recognized as an essential signal for both nitrogen fixation and denitrification.

Our intention was to explore whether cells could be pre-primed for ROS defense through modification of the single redox active cysteine (C183) in the FixK_2_ transcription factor. Our functional study of a C183D FixK_2_ variant, simulating permanent overoxidation of the protein, reveals the existence of a cellular mechanism to counteract inactivation that boosts FixK_2_ levels through transcriptional and posttranscriptional means, giving rise to wild-type phenotypes in both free-living cells and soybean bacteroids.

We believe that our research provides a platform to undertake further synthetic biology approaches to modify rhizobial FixK-type proteins and improve the durability of the symbiotic interaction and fitness in response to oxygen. This could be applied to enhance the productivity and sustainability of soybean crops, which will contribute to global food security, human health and the environment.

## Figures and Tables

**Table 1:** Symbiotic phenotype of different *B. diazoefficiens* strains on soybean plants. Shoot dry weight (SDW), nitrogen shoot content (N), nodule number per plant (NN), nodule dry weight per plant (NDW), dry weight per nodule (NDW/NN) and leghemoglobin content in nodules (Lb) were determined at 25 and at 32 days post-inoculation (dpi). WT, wild type.

Parameters	WT	Δ*fixK_2_*	C183D-*fixK_2_*
**25 dpi**			
SDW (g)	(0.54 ± 0.13)	(0.59 ± 0.10)	(0.47 ± 0.11)
N (mg)	(12.60 ± 4.0)	(4.90 ± 1.2)	(12.20 ± 3.90)
NN	(38.30 ± 4.5)	(34.50 ± 3.5)	(32.20 ± 6.70)
NDW (mg)	(38.67 ± 7.58)	(16.83 ± 1.94)	(32.50 ± 6.63)
NDW/NN (mg)	(1.03 ± 0.24)	(0.49 ± 0.03)	(1.03 ± 0.22)
Lb (mg Lb · g NFW^−1^)	(11.83 ± 0.59)	(0.11 ± 0.02)	(10.08 ± 0.35)
**32 dpi**			
SDW (g)	(0.92 ± 0.14)	(0.68 ± 0.13)	(0.77 ± 0.01)
N (mg)	(18.60 ± 5.90)	(5.00 ± 1.30)	(21.00 ± 5.80)
NN	(31.30 ± 13.60)	(50.70 ± 13.60)	(27.80 ± 4.00)
NDW (mg)	(38.17 ± 5.04)	(25.33 ± 6.31)	(32.83 ± 4.96)
NFW/NN (mg)	(1.44 ± 0.65)	(0.50 ± 0.05)	(1.21 ± 0.28)
Lb (mg Lb · g NFW^−1^)	(11.51 ± 0.24)	(0.15 ± 0.02)	(11.23 ± 0.71)

Shown are the average values ± standard deviation of one representative experiment out of at least three repetitions (*n* = 6 plants per strain at harvest point).

**Table 2:** List of the 37 genes belonging to 26 putative FixK_2_ box-associated transcription units whose expression is downregulated in both the C183D-*fixK_2_* and Δ*fixK_2_* strains in comparison to the wild type (WT), both cultured microoxically (0.5% O_2_).

Query ^a^	FC (C183D-*fixK_2_* vs. WT) ^b^	FC (Δ*fixK_2_* vs. WT) ^c^	Locus_Tag ^d^	Gene Name ^e^	Product ^f^	Position ^g^	Motif ^h^	Predicted Operon Structure ^i^
bll0330	−2.4	−11.0	Bdiaspc4_01315	-	DNA-binding response regulator	−106	TTGACCTGGATCAA	-
bll0818	−2.1	−9.3	Bdiaspc4_03880	-	hypothetical protein	−66	TTGATCCCGGTCAA	-
blr1289	−3.2	−23.1	Bdiaspc4_06390	-	oleate hydratase	−37	TTGATCCAGCGCAA	-
bll2517	−3.2	−10.2	Bdiaspc4_12930	-	acetate/propionate family kinase			-
bll2518	−2.6	−10.0	Bdiaspc4_12935	-	phosphoketolase family protein	−89	TTGACCTCACGCAA	bll2518-bll2517
bll3115	−9.6	−30.6	Bdiaspc4_16100	-	MBL fold metallo-hydrolase			-
bll3117	−2.4	−6.6	Bdiaspc4_16110	-	thymidine phosphorylase family protein	−74	ATGATCTGGGTCAA	bll3117-bll3116- bll3115
blr3815	−2.2	−7.6	Bdiaspc4_19720	-	HAD family hydrolase	−287	TTGACGTATCGCAA	-
blr4240	−3.1	−25.1	Bdiaspc4_22005	-	pyridoxamine 5’-phosphate oxidase family protein	−69	TTGAGGTGCATCAA	blr4240-blr4241
blr4241	−2.9	−83.3	Bdiaspc4_22010	-	cytochrome *c*			-
bll4412	−3.2	−20.7	Bdiaspc4_22980	-	translational machinery protein	−38	TTGACCTGCGTCAA	-
bll4634	−2.8	−20.2	Bdiaspc4_24260	-	efflux RND transporter periplasmic adaptor subunit	−75	TTGACCTAGCGCAA	-
blr4635	−2.5	−29.4	Bdiaspc4_24265	***groL5, groEL5***	chaperonin GroEL	−150	TTGCGCTAGGTCAA	-
blr4637	−2.6	−111.5	Bdiaspc4_24275	***hspC2***	Hsp20/alpha crystallin family protein	−86	TTGAGCAAAATCAA	-
bll4644	−3.2	−20.9	Bdiaspc4_24320	-	universal stress protein	−72	TTGATTTCGGTCAA	-
bll4645	−2.8	−10.6	Bdiaspc4_24325	-	host attachment protein	−69	TTGATCGGGATCAA	-
blr4652	−3.1	−95.2	Bdiaspc4_24370	-	nitroreductase	−48	TTGATCGACATCAA	blr4652-blr4653- blr4654
blr4653	−2.8	−16.8	Bdiaspc4_24375	***dnaJ***	J domain-containing protein			-
blr4654	−2.8	−30.0	Bdiaspc4_24380	-	hypothetical protein			-
blr4655	−2.5	−14.2	Bdiaspc4_24385	***ppsA***	phosphoenolpyruvate synthase	−47	TTGACCTGCCTCAA	-
bsr6066	−4.0	−92.6	Bdiaspc4_31980	-	hypothetical protein	−105	TTGACCTGTCTCAA	bsr6066-blr6067
blr6067	−2.7	−20.9	Bdiaspc4_31985	-	phage holin family protein			-
bll6073	−3.5	−27.9	Bdiaspc4_32015	***phaC2***	**probable poly-beta-hydroxybutyrate polymerase**	−81	TTGATGCAGCTCAA	-
blr6074	−2.7	−90.9	Bdiaspc4_32020	-	CBS domain-containing protein	−143	TTGAGCTGCATCAA	-
bll6525	−2.1	−7.7	Bdiaspc4_34395	-	hypothetical protein	−22	TTGATCTGCATCAA	-
bll7086	−2.3	−97.1	Bdiaspc4_37390	***hemN_2_***	oxygen-independent coproporphyrinogen III oxidase	−140	TTGCGCGAGCGCAA	-
bsr7087	−3.2	−53.8	Bdiaspc4_37395	-	hypothetical protein	−115	TTGCGCTCGCGCAA	bsr7087-blr7088
blr7088	−2.2	−8.1	Bdiaspc4_37400	-	copper chaperone PCu(A)C			-
blr7345	−2.9	−16.8	Bdiaspc4_38745	-	hypothetical protein	−76	TTGATCCGCATCAA	-
bll7986	−2.1	−5.6	Bdiaspc4_42230	-	HlyD family efflux transporter periplasmic adaptor subunit			-
bll7987	−2.5	−17.4	Bdiaspc4_42235	-	ABC transporter permease			-
bll7988	−3.3	−33.1	Bdiaspc4_42240	-	ABC transporter ATP-binding protein	−66	CTGATCTAAATCAA	bll7988-bll7987- bll7986
bll7989	−2.6	−5.3	Bdiaspc4_42245	***mat***	methionine adenosyltransferase	−203	TTGAGCCAATGCAG	-
bll7990	−3.2	−19.7	Bdiaspc4_42250	-	hypothetical protein			-
bll7991	−2.8	−22.8	Bdiaspc4_42255	-	isoprenylcysteine carboxylmethyltransferase family protein			-
bsl7992	−2.7	−23.0	Bdiaspc4_42260	-	DUF2933 domain-containing protein	−59	TTGATCTGCGTCAA	bsl7992-bll7991- bll7990
bll7993	−2.8	−8.5	Bdiaspc4_42265	-	hypothetical protein	−60	TTGAGGGATTGCAA	-

^a^ Best blast hit in the *B. diazoefficiens* USDA 110 genome ([38]; GenBank acc. # NC_004463.1; RefSeq annotation as from January 2016). Direct FixK_2_ targets as defined in [19] or validated by IVT are shaded in grey. ^b^ Fold change (FC) values of gene expression in the C183D-*fixK_2_* strain in comparison to the WT, both grown under microoxic conditions. ^c^ FC values of gene expression in cells of Δ*fixK_2_* in comparison to wild-type cells, both grown under microoxic conditions; [19]. ^d^ Nomenclature of *B. diazoefficiens* 110*spc*4 genes according to the NCBI annotation (GenBank acc. # CP032617); [30]. ^e^ Gene name according to the NCBI annotation with modifications (boldfaced) (GenBank acc. # CP032617); [30]. ^f^ Protein/gene product according to the NCBI annotation with modifications (boldfaced) (GenBank acc. # CP032617); [30]. ^g^ Position of the first nucleotide of the motif relative to the annotated translational start site of the associated gene. ^h^ Predicted putative FixK_2_ binding site. ^i^ Operon structure prediction as previously described; Ref. [19].

**Table 3:** Strains and plasmids employed in this study.

Strain or Plasmid	Description	Resistance	Source or Reference
Strains			
*E. coli*			
DH5α	*supE*44 Δ*lacU*169 (φ80 *lacZ* ΔM15) *hsdR*17 *recA1 endA1 gyrA*96 *thi*-1 *relA*1		Bethesda Research Laboratories Inc., Gaithersburg, MD, USA
S17-1	*thi pro recA hsdR hsdM* RP4Tc::Mu Km::Tn7	Tp^r^ Sm^r^ Spc^r^	[50]
ER2566	*fhuA2 lacZ::T7 gene1 [lon] ompT gal sulA11 R(mcr-73::miniTn10*-Tet^S^*)2 [dcm]* *R(zgb-210::Tn10*-Tet^S^) *endA1* Δ*(mcrC-mrr)114::IS10*		NEB, USA
*B. diazoefficiens*			
110*spc*4	Wild type (WT)	Cm^r^ Spc^r^	[51]
9043	Δ*fixK_2_*	Cm^r^ Spc^r^ Sm^r^	[26]
1255	C183D-*fixK_2_*	Cm^r^ Spc^r^	This work
3604	WT::*fixNOQP’-‘lacZ*	Cm^r^ Spc^r^ Tc^r^	[52]
9043-3603	Δ*fixK_2_*::*fixNOQP’-‘lacZ*	Cm^r^ Spc^r^ Tc^r^	This work
1255-3603	C183D-*fixK_2_*::*fixNOQP’-‘lacZ*	Cm^r^ Spc^r^ Tc^r^	This work
1109	WT:: *fixK_2_′-‘lacZ*	Cm^r^ Spc^r^ Tc^r^	[18]
9043-1109	Δ*fixK_2_*:: *fixK_2_′-‘lacZ*	Cm^r^ Spc^r^ Tc^r^	[18]
1255-1109	C183D-*fixK_2_*::*fixK_2_′-‘lacZ*	Cm^r^ Spc^r^ Tc^r^	This work
**Plasmids**			
pTXB1	Expression vector for the IMPACT protein purification system. It codes for a C-terminal thiol-cleavable *Mxe* GyrA-intein–chitin-binding domain (CBD) under T7 promoter control	Amp^r^	NEB, USA
pBBR1MCS-2	*lacPOZ mobRP4*, low-copy-number cloning vector	Km^r^	[53]
pK18*mobsacB*	Mobilizable pUC18 derivative, *mob*, *sacB*	Km^r^	[54]
pRJ0051	[pTXB1] with a 715-bp *Nde*I/*Spe*I fragment encoding C183S FixK_2_-intein fused *in frame* with the CBD of the vector		[32]
pRJ0053	[pTXB1] with a 715-bp *Nde*I/*Spe*I fragment encoding FixK_2_-Intein fused *in frame* with the CBD of the vector	Amp^r^	[32]
pRJ8848	[pUC19] with a 2.288-kb *Sal*I fragment encoding C183S FixK_2_	Amp^r^	[23]
pMB1250	[pRJ8848] with a 2.288-kb *Sal*I fragment encoding C183D FixK_2_	Amp^r^	This work
pMB1251	[pBBR1MCS-2] with a 1.843-kb *Bam*HI-*Xba*I fragment from pMB1250	Km^r^	This work
pMB1253	[pTXB1] with a 715-bp *Nde*I/*Spe*I fragment from pMB1251 encoding C183D FixK_2_	Amp^r^	This work
pRJ9041	[pUC19] with a 2.288-kb *Sal*I fragment encoding FixK_2_	Amp^r^	[33]
pMB1256	[pRJ9041] with a 3.965-kb *Not*I fragment from pMB1251	Amp^r^ Km^r^	This work
pMB1254	[pMB1256] Religation of a 4.974-kb *Bgl*II fragment	Amp^r^	This work
pMB1255	[pK18*mobsacB*] with a 1.849-kb *Bam*HI fragment from pMB1254	Km^r^	This work
pRJ3603	[pSUP202pol2] ‘blr2761, blr2762 and *fixNOQP’-‘lacZ* on a 8.261-kb *Xho*I fragment	Tc^r^	[52]
pRJ9054	[pSUP202] *fixJ*, bll2758 and *fixK_2_′-‘lacZ* on a 4.434-kb *Nsi*I/*Dra*I fragment	Tc^r^	[26]
pMB1109	[pRJ9054] *fixK_2_′-‘lacZ* with a 136-bp *Sma*I fragment deletion within the bll2758 coding region	Tc^r^	[18]

## Data Availability

Microarray data are available upon acceptance of this paper via the Gene Expression Omnibus (GEO) series record GSE196031 at the National Center for Biotechnology Information (NCBI) GEO platform (http://www.ncbi.nlm.nih.gov/geo accessed on 28 April 2022). Other additional data are provided in the Supplementary Materials.

## Supplementary Materials

The following supporting information can be downloaded at: https://www.mdpi.com/article/10.3390/ijms23095117/s1.
