# Supplementary material for: Fine-Tuning Modulation of Oxidation-Mediated Posttranslational Control of Bradyrhizobium diazoefficiens FixK2 Transcription Factor
Source: Int J Mol Sci. 2022 May 4;23(9):5117. doi: 10.3390/ijms23095117 (PMC9104804; doi:10.3390/ijms23095117)
Supplement: Supplementary file 1 [file ijms-23-05117-s001.zip › Parejo_et_al_Legends_to_Supplementary_Figures_and_Tables.pdf]

International Journal of
Molecular Sciences

MDPI

Parejo *et al.*, Legends to Supplementary Figures and Tables

Figure S1. SDS-PAGE analysis of $FixK_2$, C183S $FixK_2$, and C183D $FixK_2$ recombinant purified proteins. Samples of representative steps during protein overexpression and purification were monitored in Coomassie blue-stained 14% SDS-PAGE gels. Each panel corresponds to different sections of the same gel (C183S $FixK_2$, and C183D $FixK_2$) or a different gel ($FixK_2$). Extracts of uninduced (lanes 1, 5, and 9; 10 μL) and induced (lanes 2, 6, and 10; 10 μL) *E. coli* overexpressing cells. Purified proteins after cleavage with DTT (lanes 3, 7, and 11; ~6.25-9.2 μg), and after buffer exchange in elution buffer (40 mM Tris-HCl, pH 7.0, 150 mM KCl, 0.1 mM EDTA) (lanes 4, 8, and 12; ~3-3.5 μg). The predicted molecular masses of the purified $FixK_2$ protein derivatives (~25,6 kDa) as well as of the C-terminally bound *Mxe* GyrA-Intein-CBD recombinant protein variants (~53.4 kDa) are shown on the right margin. The molecular marker Precision Plus Protein™ Dual Color Standards (Bio-Rad, California, CA, USA) (M) is shown on the left margin.

Figure S2. Calibration curve for SEC. The following non-interacting standards were applied: In red, conalbumin (CO, 75 kDa), carbonic anhydrase (CA, 29 kDa), ribonuclease A (R, 13.7 kDa); In blue, ovalbumin (O, 43 kDa), aprotinin (A, 6.5 kDa). In black, the void volume (V_o) of was determined as 7 mL using blue dextran 2000 (BD, 2,000 kDa). Chromatograms were obtained at 0.75 mL/min using a Superdex 200 10/300 GL column with a volume (V_c) of 24 mL. The calibration curve plot of K_{av} vs. M_r (Log_{10} scale) obtained using elution volume (V_e) of each standard is inset.

Table S1. Compilation of microarray data analyses performed in this study. (Datasheet A) 104 genes differentially expressed genes in the C183D-*fixK*$_2$ strain in comparison with the wild type (WT), both cultivated under microoxic conditions (0.5% O_2). (Datasheet B) List of 54 genes that showed a differential expression in the C183D-*fixK*$_2$ strain but not in the Δ*fixK*$_2$ strain in comparison with the WT, all cultivated microoxically. (Datasheet C) List of 50 genes that showed a differential expression in both the C183D-*fixK*$_2$ and the Δ*fixK*$_2$ strains in comparison with the WT, all cultivated under microoxic conditions. The "Overview" sheet provides explanations to the individual gene groups listed in Datasheets A-C as well as the associated references.

Table S2. List of oligonucleotides used in this work.
