# Supplementary material for: Fine-Tuning Modulation of Oxidation-Mediated Posttranslational Control of Bradyrhizobium diazoefficiens FixK2 Transcription Factor
Source: Int J Mol Sci. 2022 May 4;23(9):5117. doi: 10.3390/ijms23095117 (PMC9104804; doi:10.3390/ijms23095117)
Supplement: Supplementary file 1 [file ijms-23-05117-s001.zip › Parejo_et_Table_S2.pdf]

International Journal of *Molecular Sciences*

MDPI

Table S2. List of oligonucleotides used in this work.

Primer name	DNA sequence (5′ → 3′) [(a,b,c)]	Source or reference
**Strains and plasmids verification**		
fixK2_3	GGGCATGACCTACGGGGTTC	Laboratory collection
fixK2_4	GACGTGTTCGGCCTCGAATC	Laboratory collection
fixK2_5	GAGGCTCGAGCGTTTCAC	Laboratory collection
fixK2_8	CTTTGTGGTCGATGATGACG	Laboratory collection
fixK2_9	GTCTTCCTGGTCACGTTCCT	Laboratory collection
fixK2_14	TGCGCAGCTACAAGCTTCTC	Laboratory collection
fixK2_16	TGCAGCATGATCGAGGTCAG	Laboratory collection
M13for	GTAAAACGACGGCCAGT	Universal primer
M13rev	AACAGCTATGACCATGATTACG	Longer M13 reverse primer, laboratory collection
Mf5	CGGAATTCCTGGATCATGGCGTACCTCT	Laboratory collection
Mf8	CCCAAGCTTGTCTGCATGACGTCGTTGAC	Laboratory collection
PK18_4	CTGCGCAACTGTTGGGAAGG	Laboratory collection
T3	AATTAACCCTCACTAAAGGG	Universal primer
**Site-directed mutagenesis on *fixK*$_2$**		
fixK2_mut59	GATGGCGCTGCCGATGG**A**CCGCCGCGATATCGGCG	This work
fixK2_mut60	CGCCGATATCGCGGCGG**T**CCATCGGCAGCGCCATC	This work
**FixK$_2$ derivatives expression plasmids**		
fixK2_mut19	GCGCATATGCTGACCCAGACAC	[1]
fixK2_mut58	GACTAG[TGCATCTCCCGTGATGCA]$_{intein}$GGCGTCGAGATTGTGCAGGC	[2]
**EMSA experiments**		
fixN_29_for	CGCGAAAGCCTCACATTAACAG	This work
fixN_29_rev	AAGAACACAGCGCCAGAAAC	This work
**SPR experiments**		
fixN_3_for	CCACCTATCTTGATTTCAATCAATTCCCCG	[3]
fixN_3_rev-biot	[Btn]CGGGGAATTGATTGAAATCAAGATAGGTGG	[2]

[a] Artificial restriction sites are underlined.

[b] Mutations are shown in boldface letters.

[c] Additional sequences or labels are indicated in the brackets. Btn, biotine.

References

1. Bonnet, M.; Kurz, M.; Mesa, S.; Briand, C.; Hennecke, H.; Grütter, M.G. The structure of *Bradyrhizobium japonicum* transcription factor FixK$_2$ unveils sites of DNA binding and oxidation. *J. Biol. Chem.* **2013**, *288*, 14238–14246.
2. Cabrera, J.J.; Jiménez-Leiva, A.; Tomás-Gallardo, L.; Parejo, S.; Casado, S.; Torres, M.J.; Bedmar, E.J.; Delgado, M.J.; Mesa, S. Dissection of FixK$_2$ protein-DNA interaction unveils new insights into *Bradyrhizobium diazoefficiens* lifestyles control. *Environ. Microbiol.* **2021**, 23, 6194–6209.
3. Bonnet, M. Biochemical studies on FixK$_2$, a global regulatory protein from *Bradyrhizobium japonicum*: Proteolytic control and attempts at crystallization. Doctoral Thesis, ETH-Zürich, Zürich, Switzerland, 2011. doi: 10.3929/ethz-a-006688030.
