# Supplementary figures and images for: Fine-Tuning Modulation of Oxidation-Mediated Posttranslational Control of Bradyrhizobium diazoefficiens FixK2 Transcription Factor
Source: Int J Mol Sci. 2022 May 4;23(9):5117. doi: 10.3390/ijms23095117 (PMC9104804; doi:10.3390/ijms23095117)

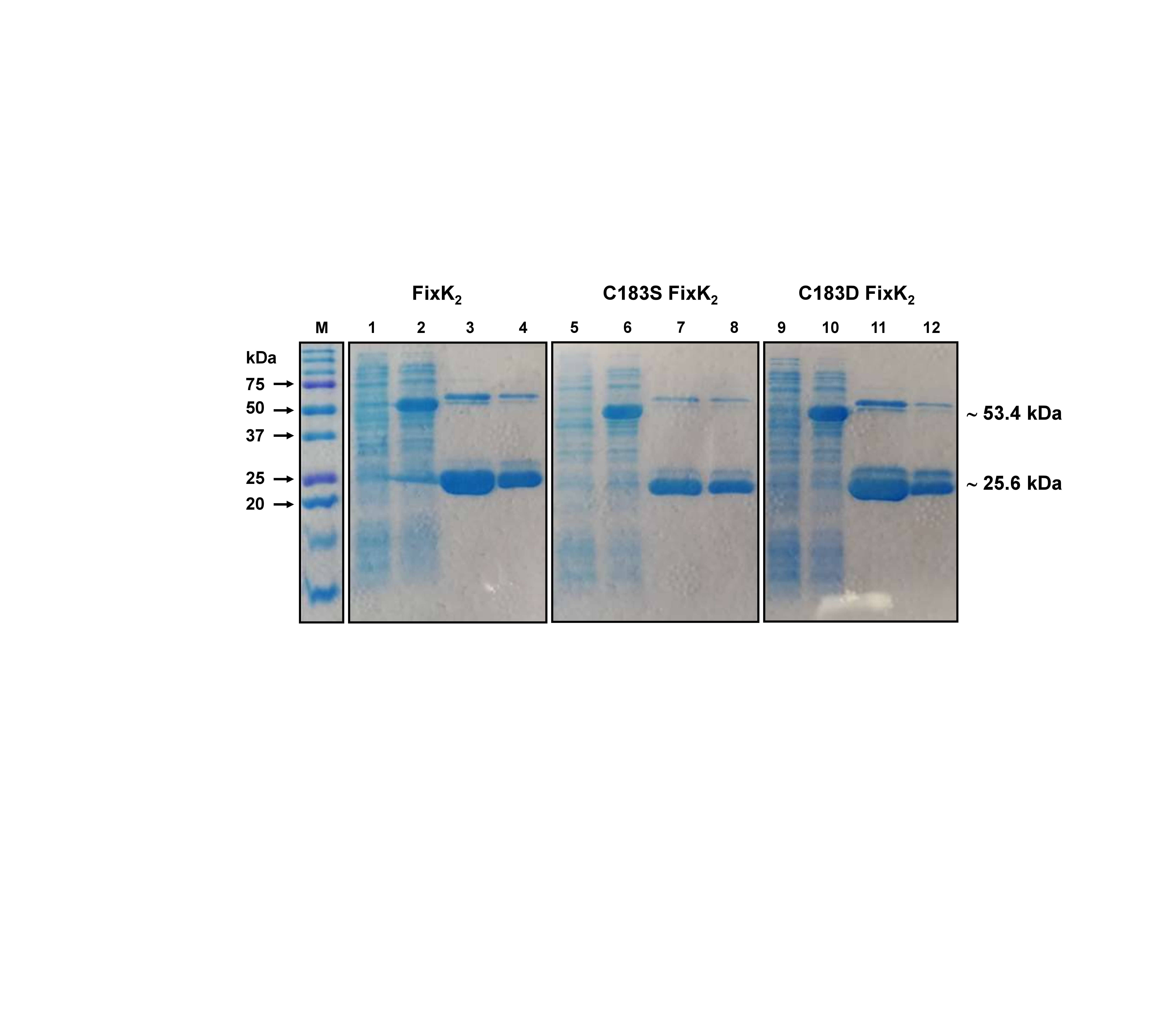

Supplement: Supplementary file 1 [file ijms-23-05117-s001.zip › Parejo_et_al_Figure_S1.tiff]
